# Virtual Interviewing and Diversity Among Psychiatry Residents

**DOI:** 10.1007/s40596-024-01999-3

**Published:** 2024-06-25

**Authors:** Mohammad Lesanpezeshki, Lincoln Chifamba, Hannah Haynie, Natalie Bonfine, Randon S. Welton

**Affiliations:** 1 Case Western Reserve University, Cleveland, OH USA; 2 Northeast Ohio Medical University, Rootstown, OH USA; 3 Kent State University, Kent, OH USA

**Keywords:** Psychiatry residency, Virtual interviewing, Diversity

## Abstract

**Objective:**

Psychiatry residency program directors were surveyed regarding their impression of the impact virtual interviewing had on the perceived and actual diversity of individuals selected for interviews and residency training.

**Methods:**

A link to an anonymous survey was sent to 299 psychiatry residencies. Psychiatry program directors provided information about their programs and rated their perception of the impact of virtual interviewing on candidates they interviewed and matched. They also reported the demographic information of incoming residency classes for 2020–2023. This data was assessed for differences in the average number of residents by each diversity category and interview format (i.e., in person or virtual) and between diversity categories and cohorts. A linear trend analysis assessed whether the number of residents in each demographic category had a significant change over time.

**Results:**

Sixty-five program directors (21.7%) provided at least partial data. Half of the responding program directors believed that virtual interviewing had increased the diversity of interviewed applicants, but there were no statistically significant differences in the average number of incoming residents who were identified as women or were in an Underrepresented in Medicine category when comparing the in-person interview year (2020) and the virtual interview years (2021–2023).

**Conclusions:**

The analyzed data demonstrated that the type of interview (in-person vs virtual) did not appear to affect the diversity of incoming psychiatry residents. Ongoing efforts to increase diversity, equity, inclusion, and belonging should be paired with measurements of their impact.

Opportunities for psychiatry residency training have dramatically increased over the last decade. Between 2012–2013 and 2022–2023, there was a 65% increase in the number of ACGME psychiatry residency programs and a 54% increase in residency training slots [1, 2]. During that time, interest in psychiatry among US medical graduates (MDs and DOs) has also grown with a 108% increase in the number of US medical graduates matching into allopathic psychiatry residencies [3, 4]. Programs were adapting to this increased interest in psychiatry when, in the spring of 2020, the COVID-19 pandemic forced psychiatry residencies within the USA to convert from in-person interviewing of applicants to virtual interviewing. With the risk and uncertainty created by the pandemic, the transition to virtual interviewing was seen as a health and safety precaution. Eventually, as the nation adapted to COVID-19, the rationale for continuing virtual interviewing shifted to cost savings and the promotion of equity among applicants [5] as it was estimated that the average US applicant was spending $3000–$4000 during interview season on travel and hotel costs [6, 7].

The initial responses to virtual interviewing were mixed. Benefits of virtual interviewing for applicants included decreasing the amount of time and money spent on traveling, increasing flexibility for interview dates, and enabling a greater number of applications [6, 8] while maintaining an adequate interview experience [9]. Virtual interviewing was more efficient for residencies as they no longer had to host applicants and interviews could be conducted from faculty members’ worksites [6, 10, 11]. Many residency programs reported that virtual interviews were effective in creating a comfortable setting and allowing them to answer interviewee questions, establish a sense of connection with the candidate, evaluate interviewee strengths, and convey the program’s culture [6, 11, 12]. Concerns about virtual interviewing included the technical challenges involved, the potential for distractions to disrupt the virtual interview, and the ability to assess applicants’ nonverbal skills and interest in specific residency programs [6–8, 11, 12].

Although there was the hope that virtual interviewing, by being more equitable, might increase the diversity of applicants who were interviewed by and subsequently matched into residency programs, it does not appear to have occurred. One aspect of diversity is “geographic diversity,” the size of a program’s catchment area. This can be assessed by measuring the distance separating the applicant’s address from the residency program. A study of five family medicine programs over 5 years, including both in-person and virtual interviewing years, found that there was no increase in geographic diversity among US applicants to these residencies or in the US applicants interviewed by the residencies [13]. An assessment of a single pediatric program receiving applications from over half of the U.S. M.D. graduates interested in pediatrics found that when excluding couples matches, there was a decrease in geographic diversity once they shifted to virtual interviewing [14].

Although some program directors reported a general sense that they were engaging with more diverse applicants through virtual interviewing [10], the actual impact on Underrepresented in Medicine (URiM) groups was unclear. URiM groups include African American, Alaska Native, American Indian, Hispanic, Latino and Native Hawaiian, and other Pacific Islander students [15]. There were concerns that these groups might be less likely than White applicants to have high-quality video equipment or stable, high-speed Internet access. Historically, many students from URiM categories have used away rotations and pre-interview social gatherings to showcase their achievements and potential. Those interpersonal interactions help them overcome factors like United States Medical Licensing Examination scores and Alpha Omega Alpha Honor Medical Society membership which disadvantage many in those categories [16, 17].

Psychiatry has tended to attract relatively high numbers of women and URiM students. A report of trainees from 2015 to 2022 found that 49.1% of US psychiatry residents were women and 12.4% were from URiM categories compared to national averages of 39.9% and 9.7% respectively [15]. Another significant source of diversity among incoming psychiatry residents has been found in International Medical Graduates (IMGs). In 2012–2013, 32% of psychiatry residency slots were filled by IMGs. As interest in psychiatry increased among US medical graduates, however, training opportunities for IMGs have decreased falling to only 18% of incoming residents in 2022–2023 [1, 2]. The impact of virtual interviewing on diversity among US psychiatry residencies is unknown. This study was designed to assess the impact virtual interviewing has had on the diversity of candidates who were interviewed and matched into US psychiatry residency training programs between 2020 and 2023. We also examined the impact of virtual interviewing on the interviewing and matching of USIMGs, US citizens trained at a medical school outside of the USA/Canada, and non-USIMGs, non-US citizens trained at a medical school outside of the USA/Canada. While appreciating the motivation to increase diversity and equity, we believe that virtual interviewing has had a limited impact on increasing the diversity in US psychiatry residencies.

## Method

We obtained email addresses for 303 of the 304 psychiatry residency programs listed by the Accreditation Council for Graduate Medical Education. An email inviting program directors to participate in our study was sent to their e-mail address or the general residency e-mail if the program director’s email was unavailable. Two more invitations to complete the survey were sent to all of the programs at weekly intervals.

The survey asked for some general information about the program: their American Psychiatric Association (APA)/American Association of Directors of Psychiatry Residency Training (AADPRT) region, demographic location of their program (e.g., rural, urban, suburban setting), and their affiliation with their sponsoring institution.

The training directors were asked about their impression of the impact of virtual interviewing on the applicants who had interviewed and matched to their program. These were measured on a five-point Likert scale from “Greatly Decreased Diversity” to “Greatly Increased Diversity.”

Training directors were asked to fill in a grid for their incoming residency classes in 2020, 2021, 2022, and 2023. They were specifically asked for the total number of incoming residents and the number of incoming residents who would identify as women, Black/African American, Hispanic, American Indian/Alaskan Native/Hawaiian (AIANH), U.S. International Medical Graduates (USIMG), or non-U.S. International Medical Graduates (Non-USIMG).

We analyzed data utilizing two statistical software programs: Jeffreys’ Amazing Statistics Program (JASP) and Statistical Package for the Social Sciences (SPSS) and conducted an independent sample *t*-test to assess differences in the average number of residents by each diversity category and interview format (i.e., in person or virtual). We utilized an analysis of variance (ANOVA) to assess differences between diversity categories and cohort (year). A linear trend analysis assessed whether the number of residents in each demographic category had a significant change (increase or decrease) over time.

The Northeast Ohio Medical University Institutional Review Board designated this as Exempted Research.

## Results

Initially, 11 surveys were returned as undeliverable but alternate emails were found for seven, meaning 299 programs received a link to an online Qualtrics survey. Sixty-five programs (21.7%) opened the survey and provided at least partial data, answering some if not all of the questions (Table 1).

**Table 1:** Data about programs

Geographic region (*N* = 53)	*N* (%)
Region 1 (New England)	5 (9%)
Region 2 (New York)	7 (13%)
Region 3 (Mid-Atlantic)	8 (15%)
Region 4 (Midwest)	8 (15%)
Region 5 (Southeast)	16 (30%)
Region 6 (California)	5 (9%)
Region 7 (Far West)	4 (8%)
Location (*N* = 57)	
Urban	35 (61%)
Suburban	9 (16%)
Rural	6 (11%)
Other/combination	7 (12%)
Affiliation (*N* = 56)	
University-based	35 (63%)
Single hospital system	3 (5%)
Community-based	15 (27%)
Other	3 (5%)

Fifty-three program directors responded to a question about the impact of virtual interviewing on the applicants they interviewed. Of that group, 51% felt that virtual interviewing had no impact on the diversity of applicants they interviewed while 43% thought that it increased and 6% thought that it greatly increased the diversity of interviewed applicants. Most of these training directors (64%) felt that virtual interviewing did not have a positive impact on the diversity of candidates who had matched into their programs while 34% expressed that it increased and 2% said that it greatly increased the diversity of residents who had matched with their programs.

Thirty-nine programs provided data for their incoming classes, but as some of these were new programs there was data for 36 programs in 2020, 37 programs in 2021, 38 programs in 2022, and 39 programs in 2023. The incoming class of 2020 was interviewed during the 2019–2020 interviewing season when interviews were almost exclusively in-person. The incoming classes of 2021, 2022, and 2023 were based on virtual interviews (Table 2).

**Table 2:** Incoming psychiatry residents by year

	2020 (In-person)	2021 (Virtual)	2022 (Virtual)	2023 (Virtual)
Programs responding	36	37	38	39
Total residents in incoming class	286	292	303	321
Women residents	164 (57%)	163 (56%)	158 (52%)	167 (52%)
Programs with at least one incoming woman resident	36 (100%)	37 (100%)	38 (100%)	38 (97%)
Black/African American residents	34 (12%)	24 (8%)	28 (9%)	24 (7%)
Programs with at least one incoming Black/African American resident	18 (50%)	16 (43%)	19 (50%)	17 (44%)
Hispanic residents	28 (10%)	30 (10%)	34 (11%)	33 (10%)
Programs with at least one incoming Hispanic resident	18 (50%)	20 (54%)	22 (58%)	20 (51%)
AIANH residents	2 (1%)	2 (1%)	3 (1%)	4 (1%)
Programs with at least one incoming AIANH resident	2 (6%)	2 (5%)	3 (8%)	4 (10%)
Total racial/ethnic URiM residents	64 (22%)	56 (19%)	65 (21%)	61 (19%)
Programs with at least one incoming racial/ethnic URiM resident	29 (81%)	28 (76%)	29 (76%)	28 (72%)
USIMG	14 (5%)	10 (3%)	10 (3%)	8 (2%)
Programs with at least one incoming USIMG resident	10 (28%)	8 (22%)	6 (16%)	6 (15%)
Non-USIMG	9 (3%)	9 (3%)	16 (5%)	14 (4%)
Programs with at least one incoming non-USIMG resident	6 (17%)	6 (16%)	13 (34%)	9 (23%)

AIANH = American Indian, Alaska Native, Hawaiian. Racial and Ethnic URiM included all residents identified as Black/African American, Hispanic, American Indian, Alaska Native, or Hawaiian. USIMG = US citizen attending an international medical school. Non-USIMG = A graduate from an international medical school who is not a US citizen

Between 2020 and 2023, the percentage of students from all URiM categories starting in this sample of residency programs declined slightly from 22 to 19%, while the percentage of programs with at least one incoming resident from a URiM category declined from 81 to 72%. These changes were not statistically significant.

Comparing the in-person interview year (2020) with the virtual interview years (2021–2023) using an analysis of variance (ANOVA) with linear trend analysis indicated that there is no significant difference in the average number of women by cohort (*p* = 0.995). There were also no statistical differences in race/ethnicity categories by cohort for Hispanic residents (*p* = 0.905), AIANH residents (*p* = 0.787), Black/African American residents (*p* = 0.641), or overall URiM residents (*p* = 0.905). Finally, there was also no significant difference between the numbers of incoming USIMG (*p* = 0.534) or non-USIMG (*p* = 0.554) residents.

## Discussion

Between 2020 and 2023, there was a shift from in-person to virtual interviewing of applicants to psychiatry residency programs. We examined both the impressions of residency program directors and program-level data regarding their incoming residents. Half of the responding training directors felt that virtual interviewing increased the diversity of their interviewed applicants while most did not believe that it increased the diversity of the incoming residents.

Our analysis of incoming residents found no significant difference in any of the diversity categories (i.e., race/ethnicity, gender, or international medical school graduates) by interview method (in-person or virtual) or by year of cohort.

For the survey respondents, it does not appear that the format of the interview meaningfully affected the diversity of their incoming general psychiatry residency class. These non-significant findings align with the perceptions of training directors who felt that virtual interviewing did not impact the diversity of the incoming class of psychiatry residents. One-third of the training directors believed that the virtual interview format resulted in an increase in diversity in incoming resident classes, but we were unable to demonstrate that change.

There are numerous limitations to this study. There was a low response rate, and many program directors did not complete the entire survey. As some of the residency programs started accepting residents during the timeframe covered by the survey, those program directors would have a limited ability to assess the comparative impact of virtual interviewing on the diversity of their applicants. It is unclear, therefore, if our results can be generalized to all psychiatry residency programs, and future research should explore the trends noted here. There is also the chance that because we looked at aggregated data, we failed to discern differences among different types of residency programs. For example, there may be meaningful differences depending on the location of the residency (urban vs rural), type of program (community-based vs hospital-based), or region of the country. Our study did not have the statistical power to discern those trends. The data were also impacted by the fact that social concerns across the country occurring at this same time led many programs to engage in a variety of approaches and programs to increase diversity, equity, inclusivity, and belonging (DEIB) among residents. These efforts occurred simultaneously with the shift to virtual interviewing and with growing numbers of US medical graduates applying to psychiatry residency programs. The interaction between virtual interviewing, this increased interest in psychiatric residency training, and these DEIB efforts and their cumulative impact on applicants must be highly complicated and nuanced, but they are outside the scope of the current analysis. Further research could focus on why a perceived increase in the diversity of applicants who were interviewed did not result in an increase in the diversity of individuals matched into these residency programs.

The decision to interview resident applicants in person or virtually does not appear to affect the diversity of incoming psychiatry residents. Our data indicated that it did not harm but may not have increased the diversity of applicants who were matched with psychiatry residency programs. Whether training programs continue with current strategies attempting to impact diversity or devise new ones, leaders in training and education should do so with the intention of measuring the impact of those interventions. Future efforts to increase equity, diversity, inclusivity, and belonging must be supported by data to determine the efficacy of interventions and approaches.

## Data Availability

Original data is maintained by corresponding author and can be accessed through him.
